# 24-h movement behaviour, thermal perception, thirst, and heat management strategies of children and adults during heat alerts: a pilot study

**DOI:** 10.3389/fphys.2023.1179844

**Published:** 2023-05-09

**Authors:** Nicholas Ravanelli, Nathan Morris, Shawnda A. Morrison

**Affiliations:** ^1^ School of Kinesiology, Lakehead University, Thunder Bay, ON, Canada; ^2^ Centre for Research in Occupational Safety and Health, Laurentian University, Sudbury, ON, Canada; ^3^ William J. Hybl Sports Medicine and Performance Center, Department of Human Physiology and Nutrition, University of Colorado Colorado Springs, Colorado Springs, CO, United States; ^4^ Faculty of Sport, University of Ljubljana, Ljubljana, Slovenia; ^5^ Human Potential Translational Research Programme, Yong Loo Lin School of Medicine, National University of Singapore, Singapore

**Keywords:** heat wave, thermal perception, heat mitigation strategies, cooling solutions, adults, children, thirst, physical activity

## Abstract

**Background:** Heat waves caused by climate change are increasingly challenging the wellbeing of individuals across the lifespan. Current efforts to understand the thermal perceptions and behaviours of people throughout the lifespan during heat waves are limited.

**Methods:** Since June 2021, the Active Heatwave project has been recruiting households to better understand how individuals perceive, cope, and behave during heat waves. Using our novel web platform, participants were prompted to answer our *Heat Alert Survey* on days when a participants geolocation corresponded to a broadcasted local heat alert. Participants provided 24-h movement behaviour, thirst, thermal perception, and cooling strategies via validated questionnaires.

**Results:** A total of 285 participants (118 children) from 60 distinct weather station locations globally participated between June and September 2021 and 2022. At least 1 heat alert (834 total) were identified from 95% (57/60) of the weather stations. Children reported spending more time performing vigorous intensity exercise compared to adults (*p* < 0.05), but no differences in thermal sensation, thermal comfort, or thirst sensation (all *p* > 0.31) were observed. For thirst management, 88% of respondents used water to relieve thirst, although notably, 15% of adults reported using alcohol. Regardless of age, staying indoors was the most common heat management strategy, whereas visiting cooling centres was the least.

**Conclusion:** The present study presents a proof-of-concept combining local heat alert notifications with e-questionnaires for collecting near-real-time perceptual and behavioural data for both children and adults during heat waves. The observed patterns of behaviour suggest that present public heat-health guidelines are often ignored, children engage in fewer heat management strategies compared to adults, and these disparities highlight the need to improve public health communication and knowledge dissemination around promoting effective and accessible cooling solutions for children and adults.

## Introduction

Periods of extreme heat, or heat waves, are predicted to continue increasing in terms of their frequency, intensity, and duration (Pörtner et al., 2022). Heat waves already exert a tremendous burden on emergency services and first-responders (Bassil et al., 2011; Turner et al., 2013), including elevated hospital admissions (Sun et al., 2021; Bernstein et al., 2022), and increased mortality rates amongst the most vulnerable populations (Semenza et al., 1996; Basagaña et al., 2011; Ebi et al., 2021). It is therefore imperative that policymakers, scientists, and the public better understand how ambient temperature influences thermal perception, and how their subsequent behaviour may be affected during heat waves in order to optimize current heat management strategies and ameliorate their dissemination efficacy to the public.

In comparison to adults, children have a potentially reduced capacity to cope with high ambient temperatures due to a combination of physiological, morphological, and behavioural differences (Notley et al., 2020). Younger children in particular are not always in direct control of their own movement patterns, indoor climate preferences (e.g., thermostat control), nor are they even in control of the clothing that they wear, and as a consequence, may find themselves in a position where they will be unable to physically remove themselves from a ‘thermally-threatening’ environment, especially if their caregiver doesn’t know (or notice) overt signs of heat illness. Rather, appropriate behavioural thermoregulation actions are primarily governed by a child’s guardian(s). Recent work has demonstrated that in absence of direct contact, parents (Chen et al., 2022) and caregivers (Folkerts et al., 2020) are not able to accurately assess the thermal state of children. Additionally, the National Health and Nutritional Examination Survey concluded that over 75% percent of children do not meet recommended water intake from the US Institute of Medicine (Drewnowski et al., 2013) and over 50% are underhydrated (inferred from urine osmolality (Kenney et al., 2015)). Similarly, over 80% of children in some European countries do not meet the European Food Safety Authorities’ water intake recommendations (Senterre et al., 2014; Vieux et al., 2016). When offered water ad-libitum during exercise in the heat, children may insufficiently rehydrate, further exacerbating their thermal and cardiovascular strain (Bar-Or et al., 1980). Since children may also be unable to effectively communicate their thermal status or thirst sensation to adults (Garcia and Sheehan, 2016), their direct engagement in heat management strategies is critical to consider; a child’s perception of their immediate environment, and ability to gauge the temperature of their own bodies, may also differ from adults, especially during periods of extreme heat. Previous work has found that children spend more time outdoors engaging in physical activity than their adult counterparts (Sheffield and Landrigan, 2011), even though playing outside can increase their thermal strain (McGarr et al., 2020). Thus, prolonged outdoor physical activity engagement may exacerbate a child’s risk of experiencing heat-related injuries during periods of extreme heat (Sheffield and Landrigan, 2011). Whether children actually engage in larger quantities of physical activity (or at higher intensities) compared to adults during heat waves remains unknown.

Thus far, attempts to quantify perceptual or behavioural responses have employed singular self-reported surveys that are often delivered in the absence of a heat wave (Alberini et al., 2011; Hayden et al., 2017; Soebarto et al., 2019), although some attempts have been made to correlate responses to local weather trends (Pisello et al., 2017). With the advancements in web applications, the capacity to obtain individual data when a heat alert is active for a specific region has become more accessible, but this potential has yet to be fully exploited or engaged. Thus, the purpose of this pilot study was to build a global database framework capable of evaluating the 24-h movement behaviour, thermal perception, and cooling strategies of children and adults when a local, real-time heat alert was broadcasted using a bespoke web application.

## Methods

Following ethical approval from Lakehead University Research Ethics Board, and multi-jurisdictional approval from University of Colorado Springs and University of Ljubljana, individuals were invited to participate in the Active Heatwave study by registering to an account hosted on a custom, protected web application (https://activehw.lakeheadu.ca, written and developed by NR). Recruitment mechanisms included social media posts, traditional media interactions (e.g., interviews, print media), word of mouth, and snowball sampling. The sample population of interest was any family or single adult who resided in Canada, Slovenia, or the United States. During registration, adult users provided their informed consent for themselves and all interested participating members of their household (i.e., child assent (de Zulueta, 2010)). Participants were initially stratified into two categories within the online application (children < or = 18 years, and adults > or = 19 years), however for analysis we were interested in evaluating whether different responses would be observed between pre-pubertal (≤10 years) and adolescent peers (11–18 years). They identified their primary place of residence (e.g., city, state/province, country) using either manual input or a browser-based geolocation tool. Once their account was verified, all participants supplied their age, height, and body mass through the web platform. Local weather data corresponding to their geolocation was provided by the participating users, and was monitored between June and September during 2021 and 2022 using the OpenWeatherMap Application Programming Interface (API, https://www.openweathermap.org/). In addition to local weather data, OpenWeatherMap API aggregates Weather Alerts, including Heat Advisories, directly from government agencies, including those from: Canada (Meteorological Service of Canada), Slovenia (National Meteorological Service), and the United States of America (Integrated Public Alert and Warning System, National Oceanic and Atmospheric Administration). The presence of a local heat alert was identified when specific keywords were found within the issued alert text (e.g., ‘heat’, ‘high temperature’, ‘heat wave’, and/or ‘heat warning’). When our software system detected a heat alert within a monitored region, all participants who defined this region as their primary residence were programmatically notified via email to fill out their *Heat Alert Survey*. Participants were asked to complete the survey during a heat alert, or within 24 h of it being terminated, in order to mitigate against/minimize recall bias. Participants were prompted to fill out the *Heat Alert Survey* for every day that a heat alert was issued up to and including 3 days in a row, after which the automated notifications would stop. This was done because during the first iteration of pilot data collection (summer 2021), some participants provided feedback that there were too many alert requests (e.g., 7–10 days of continuous alerts from their national weather service for their region) and they were losing interest in filling out the data every time. Participants could complete their heat alert survey for every heat alert day they experienced, however, for the purposes of this pilot assessment, only data from participants’ first submission was included in the analysis. This approach was to reduce the possibility of inflating our Type 1 error, avoid possible dilution of effects due to heat acclimation across sequential days, and minimise habituation (learning) effects.

### Heat Alert Survey

The questionnaire was identical for all participants, and fully accessible through the online platform. Children 12 years of age and under were assisted by their parent or guardian to complete the questionnaire. In order to mitigate possible parental bias, surveys for any children were completed first, then by any adults (≥19 years of age). Adults were prompted to ask the children directly any questions regarding temperature perception, and their thirst and behaviour. The questionnaire was comprised of a modified School Health Action, Planning and Evaluation System (SHAPES) questionnaire (Leatherdale et al., 2009), where recall questions were restricted to querying only the last 24 h period (versus a full 7-day recall version, See Supplementary File). The modified SHAPES questionnaire is a subjective physical activity assessment instrument which asks respondents to quantify the amount of moderate and vigorous physical activity (MVPA) completed, recreational sedentary time, and overall sleep duration. The questionnaire was back translated from English to Slovenian by two native Slovenian speakers, following World Health Organization recommendations for translation and adaptation of instruments (World Health Organization, 2009). The SHAPES questionnaire has adequate reliability and validity for the populations studied (Adamo et al., 2009). MVPA was calculated based on the sum total of the self-reported moderate and vigorous minutes. Recreational screen time was determined by summing the variables used to assess total screen time (e.g., time spent watching television, watching videos on a computer or DVD, using a cell phone, playing videogames, browsing on the internet). In terms of thermal assessments, the *Heat Alert Survey* included a 9-point thermal sensation scale, 5-point thermal comfort scale, 5-point thirst sensation scale, in addition to identifying any hydration or heat management strategies employed by the participants during their heat alert period.

### Statistical analysis

All data are presented as mean ± standard deviation. A *p*-value less than 0.05 was set as the level of significance *a priori*. A Kurskal-Wallis nonparametric test was used to compare each of the dependent variables (SHAPES questions 1-4, sleep duration, thermal sensation, thermal comfort, thirst sensation) between three groups (<10 years young children, 11–18 years adolescents, 19 + y adults) as all dependent variables violated normal distribution assessed with a Shapiro-Wilk test. Where significance was observed, *post hoc* comparisons between groups were conducted using a Dunn’s multiple comparisons test. The frequency of thirst and heat management solutions was expressed as a percentage of the group sample size. Independent Chi-squared tests were used to evaluate whether differences were observed for i) thirst and ii) heat management solutions between all three groups (<10, 11–18, 19+), and post-hoc comparisons were completed using multiple Mann-Whitney U tests. All analyses were conducted using GraphPad Prism Version 9.4.1 (La Jolla, United States).

## Results

### Survey respondents

A total of 285 participants (118 children) from 60 distinct weather station locations globally participated in the Active Heatwave study. Between the months of June and September 2021 and 2022, at least 1 heat alert (834 total) was identified from 95% (57/60) of the weather stations being monitored. Following 1295 independent telecommunication broadcast requests for participants to complete a *Heat Alert Survey*, a total of 271 heat alert surveys from 97 unique participants were submitted; Sample sizes for 10 years and under (U10, n = 18), from 11 to 18 years of age (U18 n = 13), and 19 years of age or older (19+, n = 66) were compiled. Participant characteristics are presented in Table 1.

**TABLE 1:** Mean participant characteristics (n = 97) each group (10 years and under: U10, 11–18 years of age: U18, and 19 years of age or older: 19+).

	U10 (n = 18)	U18 (n = 13)	19+ (n = 66)
Age (y)	6 ± 1	13 ± 2	42 ± 11
Sex (M/F)	10/8	4/9	25/41
Mass (kg)	19.4 ± 4.8	48.7 ± 16.5	65.6 ± 21.4
BMI	14.4 ± 2.0	18.5 ± 4.4	22.4 ± 6.3

BMI: body mass index.

### Physical activity habits, sedentary behaviour, and sleep

**FIGURE 1:**
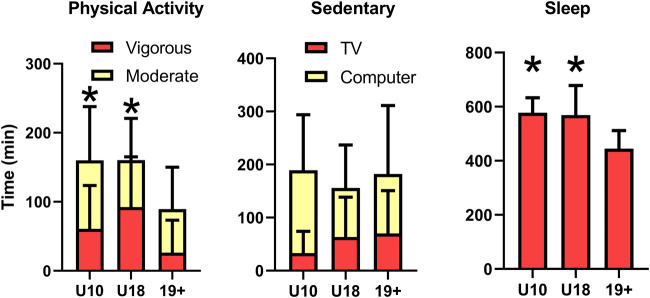
Self-reported time (mean ± standard deviation) spent engaged in moderate and vigorous physical activity (left), recreational sedentary activities (TV and Computer use, middle), and total sleep time (right) for children 10 years and under (U10), children from 11 to 18 years (U18), and adults (19+). *Time engaged in vigorous physical activity, or sleep time, is significantly greater (*p* < 0.05) than adults.

A main effect was observed for total minutes spent conducting vigorous (*p* = 0.002) but not moderate (*p* = 0.15) physical activity (Figure 1). In comparison to adults, both U10 (*p* = 0.05) and U18 (*p* = 0.007) children reported spending more minutes engaging in vigorous intensity exercise. No differences in recreational sedentary time were observed between groups (*p* > 0.18). Both U10 and U18 groups reported longer sleep duration in comparison to adults during heat alert days (*p* < 0.0001).

### Thermal perceptual responses

**FIGURE 2:**
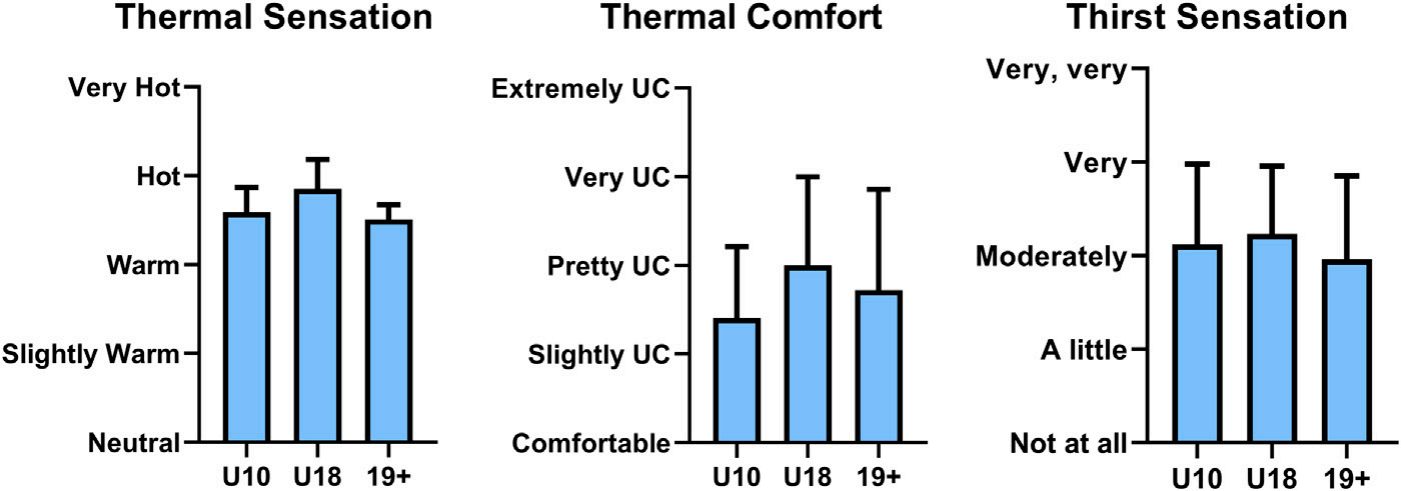
Self-reported average thermal sensation (left), thermal comfort (middle), and thirst sensation scale (right) of children 10 years and under (U10), children from 11 to 18 years (U18), and adults (19+) during a local heat alert (mean ± standard deviation). UC: Uncomfortable.

No main effects were observed between groups for thermal sensation (*p* = 0.69), thermal comfort (*p* = 0.31), and thirst sensation (*p* = 0.52, Figure 2).

### Thirst and heat management strategies

**FIGURE 3:**
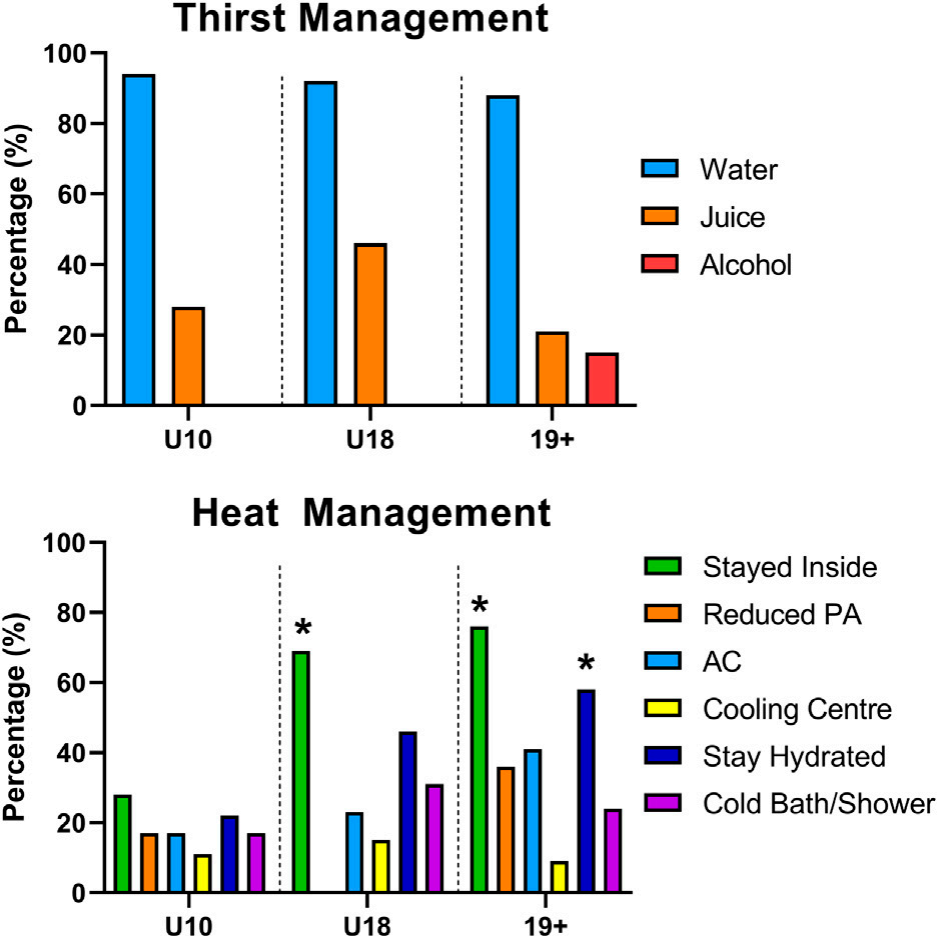
Self-reported thirst (top) and heat (bottom) management solutions of children 10 years and under (U10), children from 11 to 18 years (U18), and adults (19+) during a local heat alert. Values indicate the percentage of the group population who listed this management solution, and participants could select more than one option. *Significantly higher than U10 (*p* < 0.05).

The distribution of thirst management strategies employed by each group were significantly different, X^2^ (6, n = 97) = 47.25, *p* < 0.0001 (Figure 3A). More than 88% of all respondents for each group selected water as their first thirst management solution. Juice was the second-most reported option being selected highest among U18 (46%) followed by U10 (28%) and Adults (21%). 15% of adults surveyed selected alcohol as a thirst management solution during the heat alert period. While no U10 individual reported their thirst going away, 8% of U18% and 3% of adults reported their thirst spontaneously ‘going away’ without any particular or active intervention.

Lastly, the distribution of heat management strategies identified by each group were significantly different, X^2^ (10, n = 97) = 42.99, *p* < 0.0001 (Figure 3B). Staying indoors was the most common heat management strategy cited amongst all groups (U10: 28%, U18: 69%, Adults: 76%), although it was reported significantly less among U10 compared to U18 (*p* = 0.03) and adults (*p* < 0.001). Staying hydrated was the second-most common heat management strategy identified by all groups (U10: 22%, U18: 46%, Adults: 58%) with adults reporting this strategy significantly more than U10 (*p* = 0.02). Reducing physical activity was cited most commonly in adults (36%) compared to U10 (17%), and this strategy was not reported at all within the U18 group. A cold shower/bath was most commonly cited in U18 (31%), followed by adults (24%) and U10 (17%). Air conditioner use was most commonly identified by adults (41%), following by U18 (21%) and U10 (17%). Visiting cooling centres was the least common heat management strategy selected according to all 3 groups (U10: 11%, U18: 15%, Adults: 9%).

## Discussion

In this study, we employed a novel method of attempting to discern the physical activity habits, thermal perceptions, and thirst and heat management strategies in children and adults during heat waves, by combining local heat alerts with an automated electronic questionnaire sent directly to the participants. In comparison to adults, both U10 and U18 groups of children reported greater time spent engaged in physical activity, with no difference between groups observed for recreational sedentary time. Whereas no significant differences were observed between groups for thermal sensation, thermal comfort, or thirst sensation, thirst and heat management strategies were different between age groups during heat alert days. Collectively, this pilot study provides ‘proof-of-concept’ for an online platform designed to prompt and record perceptual responses and perceived heat-defensive actions, in response to local heat alerts, thereby reducing the recall bias typical of *post hoc* questionnaires.

Current heat health action plans recommend that individuals limit their physical activity during heat waves, and suggest avoiding physical activity during the hottest periods of the day (Singh et al., 2019; Canada, 2022). However, the expected increases in frequency, duration, and intensity of heat waves globally (Pörtner et al., 2022) will limit an individual’s ability to safely and consistently meet the World Health Organization guidelines that state all persons should be performing at least 30–60 min of physical activity on average per day (World Health Organization, 2020). The recent Lancet Countdown Report on Health and Climate Change (Romanello et al., 2022) estimated that between 2012–2021 relative to 1991–2000, the number of annual hours of moderate and high-heat stress risk during light outdoor physical activity rose by 33% and 44%, respectively. Additionally, with nearly 20% of children globally not meeting these guidelines (Tapia-Serrano et al., 2022), it remains that the epidemic of physical inactivity continues to be an urgent public health concern, and consistently hotter weather may further reduce the likelihood of physical activity engagement (Heaney et al., 2019; An et al., 2020). Promisingly, in comparison to adults, both the U10 and U18 groups reported engaging in nearly 2-fold greater MVPA during heat alerts days. This was surprising since hotter weather and other weather-related events can adversely affect total physical activity time in children (Lewis et al., 2016). In the present study, we found that U10 individuals were least likely to report “staying inside” as a heat management solution with the implication that physical activity occurred in combination with ambient heat stress from playing outdoors which may increase their risk of exertional heat illness. Future work is warranted to better identify whether individuals continue to engage in physical activity with or without added environmental heat stress (i.e., do they opt to exercise indoors or in climate conditioned spaces more frequently?). Nevertheless, heat health action plans should include and/or further promote how individuals can continue to safely engage in the recommended 30–60 min of daily physical activity (World Health Organization, 2020) during periods of extreme heat.

All age groups reported engaging in >2 h recreational screen time, exceeding the WHO guidelines on sedentary behaviour which suggests limiting recreational screen times to a maximum of 2 h per day or less, especially for children (World Health Organization, 2020). Higher ambient temperatures have been associated with increased screen time and sedentary behaviour in children (Lewis et al., 2016). Whether the current self-reported screen time and sedentary behaviours during heat alerts are different during non-heat alert days remains unknown. The amount of nocturnal sleep recommended for children 6–12 years of age is suggested to be between 9 and 12 h per 24 h, and for teenagers 13–18 years of age, sleep times should consistently range between 8 and 10 h per 24 h to promote optimal health (Paruthi et al., 2016). Adults should sleep a minimum of 7 h (or more) on a regular basis (Watson et al., 2015). On average, all populations reported meeting these minimum consensus standards, even during heat alert days.

In terms of one’s perception to thermal stress, research has shown that children have different neutral temperature limits compared to adults (Teli et al., 2013; Chen et al., 2022), however direct comparisons between children and adults for the same environmental condition are limited. Furthermore, most assessments of children’s thermal perceptions have been conducted within classroom settings (Teli et al., 2012; Teli et al., 2013), which are often air-conditioned, during rest, and not externally-valid to outdoor play scenarios. To our knowledge, only one study has reported the thermal perceptions between children and adults; data were gathered during a concurrent 30-min exposure protocol conducted in a climate chamber (Chen et al., 2022). Chen et al. (Chen et al., 2022) reported a wider and lower neutral temperature zone within preschool children compared to adults. The children’s mean ‘neutral’ temperature was approximately 2.2°C lower than their parents (20.1°C versus 22.3°C). The upper range of neutrality remained similar between children and their parents, suggesting children may experience similar thermal perceptions with increasing temperature compared to adults. This pilot study also found similar thermal sensation and comfort levels between children and adults during heat alert days.

A difference in cooling strategies between groups was observed in the present study (Figure 3). Notably, U10 consistently engaged in fewer cooling strategies relative to U18 and 19+, despite reporting similar thermal sensation and comfort (Figure 2). Whether children require fewer cooling interventions during heat waves relative to adults to maintain similar thermal comfort or sensation remains unclear. Alternatively, children may not recognize specific activities as heat mitigation strategies (i.e., staying indoors or reducing physical activity) which further emphasizes the need for accessible educational resources for children to increase awareness of primary thermal strain prevention strategies. Next, the most underutilized cooling strategy reported was visiting a cooling centre, a prominent cooling solution in heat health guidelines (Singh et al., 2019; Canada, 2022). A recent Centers for Disease Control and Prevention report (Widerynski et al., 2017) identified barriers to accessing or using cooling centres include, but are not limited to, knowledge of their location, inability to leave home, limited access to transportation, and negative association of cooling centres for strictly vulnerable populations. Nevertheless, more than 50% of the population in 3 major metropolitans in Canada are within a 15-min walk to a defined cooling centre (Quick et al., 2022), and up to 63% of cooling centres in 81 US cities are within a 0.5-mile radius of ones residence (Adams et al., 2023). No data currently exists to describe the prevalence and accessibility of cooling centres in Slovenia. While climate conditioning to facilitate heat avoidance will undoubtedly reduce thermal strain for visitors, maintaining cooling centres during heat waves places excessive strain on power grids causing instability and possible power failures, which can further exacerbate negative health outcomes in the community (Anderson and Bell, 2012), second to increasing greenhouse gas emissions (Barnett and O’Neill, 2010). Thus, reliance on cooling centres as a primary heat management strategy by heat health guidelines should be reconsidered, and more efficient heat management solutions should be promoted by public agencies.

Next, nearly 1/5 adults reported consuming alcohol as a ‘rehydration strategy’ during heat alert days, which is concerning as alcohol consumption has been demonstrated to be one of the more prevalent comorbidities for heat stroke during heatwaves (Dematte et al., 1998). Presently, it is unclear how alcohol consumption contributes to heat-related illness as some studies suggest that alcohol consumption does not modulate temperature regulation in humans during heat stress (Desruelle et al., 1996; Yoda et al., 2005). Alternative explanations include increased diuresis leading to dehydration (Wiese et al., 2000), although a recent randomized trial observed no acute difference in hydration status with lager beer consumption relative to still water (Maughan et al., 2016), and impaired decision making (Bechara et al., 2001). Further, excess alcohol consumption during heat waves may increase one’s risk of negative health consequences (Kilbourne et al., 1982) or exacerbate violent behaviour (Bulbena et al., 2009). Therefore, although the common recommendation to avoid alcohol consumption during hot weather events (Organization, 2011) is likely a sound one, the present results suggests it is being ignored. Future studies are warranted to investigate whether these negative health consequences are associated with low-to moderate-consumption of alcoholic beverages during periods of extreme heat, and whether adherence to public health guidelines can be improved.

### Methodological innovation

Studies attempting to evaluate individual heat mitigation strategies and perceptions of extreme heat events typically employ focus groups (Hansen et al., 2011; Lao et al., 2016), interviews (Wolf et al., 2010; Kemen et al., 2021), or questionnaires completed in-person (Lam et al., 2018), by telephone (Kosatsky et al., 2009; Nitschke et al., 2013), or online (Pisello et al., 2017). Unfortunately, responses received in these aforementioned protocols often do not occur during an actual heat wave, or require researchers to mobilize data collection efforts during periods of elevated ambient temperatures. To our knowledge, only one study has attempted to sample citizen responses during periods of the year where heat waves were more prevalent using an online survey (Pisello et al., 2017). However, they did not specifically target responses during a heat alert, but rather, categorized anonymous responses received during the month of August and expressed them relative to when a heat wave occurred. The current study leveraged a custom web application developed by the research team to automatically monitor the local weather of participants for heat alerts broadcasted by local government agencies, and programmatically issue a prompt to complete a *Heat Alert Survey* within 24 h. This innovative approach enabled a highly efficient *in situ* survey distribution, limited recall bias, supported multiple languages, and promoted data governance by the research team to aggregate physical activity habits, thermal perceptions, and cooling strategies from children and adults. This study served to validate our methodological approach, and we therefore invite researchers to participate in future iterations to more comprehensively understand how behavioural responses and thermal perceptions change during heat waves at a global scale.

### Limitations and future considerations

The present pilot study is not without its limitations. The small sample size may limit the conclusions drawn and thus further work is warranted among a larger heterogenous population. While we attempted to characterize day-to-day changes by permitting participants to continue to complete *Heat Alert Surveys* for each day a heat alert was active, a relatively small percentage of participants continued to submit responses on successive heat alert days limiting interpretation. Further, our small sample among the elderly population (+65 years, n = 3) limited a 4th group for comparisons, warranting future work. Interpretations of physical activity levels between children and adults are based on self-reported recall data. Although the SHAPES questionnaire has been validated (Adamo et al., 2009), future work should consider objectively measuring physical activity levels using commercially available activity monitors to more comprehensively understand how heat alerts may, or may not, impact actual physical activity habits done during daily life. Also, movement restrictions associated with the COVID-19 pandemic may have influenced the daily physical activity habits of participants, thereby not truly reflecting the ‘normal’ activity engagement. Canada (Colley and Watt, 2022), United States (Pfefferbaum and Van Horn, 2022), and Slovenia (Morrison et al., 2021) have each reported reduced physical activity habits among children throughout the COVID-19 pandemic. We must therefore stress that the preliminary duration of physical activity reported in this study should be confirmed with objectively-measured physical activity tools in the future. Local heat alert monitoring was handled by a third-party data aggregation platform (OpenWeatherMap) which collates weather alerts from various government agencies. Heat warning thresholds are not universally defined or standardised between countries. In order to mitigate this issue, we only requested survey completion from participants when a local heat alert was broadcasted in their area to ensure comparable, region-specific conditions. Adults and children were presented the same survey questions, and as such, there is the potential for literacy limitations or bias among children in how they would be interpreting their own thermal sensation and comfort. Lastly, whilst our survey did permit some speculation regarding the severity of outdoor heat exposure between groups, it remains unknown the exact amount of heat exposure participants actually experienced during their broadcasted heat alert.

### Perspectives

Vulnerable populations such as the elderly, those with comorbidities, outdoor occupational workers, and children are at an increased risk of heat-related complications during periods of extreme heat (Ebi et al., 2021). Behavioural temperature regulation may play a critical role in initiating heat relief strategies during heat waves, working alongside the specific physiological mechanisms to that target population when attempting to mitigate adverse heat health outcomes. Whereas age-related alterations in thermal perception have been identified in adults (Takeda et al., 2016; Baquero and Forcada, 2022), there is limited evidence available for children and adolescents. The present study demonstrates that self-reported thermal sensation and comfort are similar between children and adults during periods when a local heat alert is broadcasted. Children under the age of 10 reported engaging in fewer heat management strategies compared to U18 and adults, whilst maintaining higher physical activity habits within their 24 h movement behaviours. These findings support the need for future work to disseminate clear, effective and targeted messaging when educating adults to recognize the signs and symptoms of overt heat illness for themselves and also their children, which incorporate proper behavioural thermoregulation actions when temperatures rise. Long term heat health risk solutions are required to balance the need to maintain adequate lifelong physical activity habits in the context of a warming world.

## Conclusion

During local heat alert days, children spend more time engaged in vigorous, but not moderate, physical activity compared to adults. Thermal sensation, thermal comfort, and thirst sensation did not differ between children and adults. Thirst and heat management strategies differed between children and adults, with children engaging in fewer heat management strategies compared to adults, highlighting the need to create more effective public health communication and knowledge dissemination promoting effective and accessible cooling solutions to both parents and children.

## Data Availability

The raw data supporting the conclusion of this article will be made available by the authors, without undue reservation.
