# Supplementary material for: 24-h movement behaviour, thermal perception, thirst, and heat management strategies of children and adults during heat alerts: a pilot study
Source: Front Physiol. 2023 May 9;14:1179844. doi: 10.3389/fphys.2023.1179844 (PMC10203601; doi:10.3389/fphys.2023.1179844)
Supplement: Supplementary file 1 [file DataSheet1.DOCX]

**Active Heatwave – Heat Alert Survey**

**1:** Indicate how many minutes you have been engaged in STRONG physical activities (where you have an increased heart rate, shortness of breath and sweating) on the day identified in Question 1. This includes physical activity during sports / physical education, during breaks, after school, in the afternoon, in the evening and at leisure.

*For example:* If you have been practicing for two hours, you must mark the number 2 for the hours and 0 for the minutes.

Hours: _____ Minutes: ______

**2:** Indicate how many minutes you have been doing MODERATE physical activities (slightly out of breath) on the day identified in Question 1. This includes physically active arrival and departure from school, physical activity during sports / physical education, during breaks, after school, in the afternoon, in the evening and at leisure.

*For example:* if you did 1 hour and 30 minutes of moderate physical activity, mark the appropriate fields separately in the hour table and separately in the minute table

Hours: _____ Minutes: ______

**Question 5:** Indicate how many minutes you have watched a TV program or movies via DVD or the Internet on the day identified in Question 1.

Hours: _____ Minutes: ______

**2:** Indicate how many minutes you have spent on your computer or entertainment tablet, e.g. played games, browsed the internet on the day identified in Question 1.

Hours: _____ Minutes: ______

**3:** What time did you wake up today?

_______________

**4:** What time did you fall asleep last night?

_______________

**5:** How would you rate your overall thermal comfort on the day identified in Question 1?

- Extremely Uncomfortable
- Very Uncomfortable
- Pretty Uncomfortable
- Slightly Uncomfortable
- Comfortable

**6:** How would you rate your overall thermal sensation on the day identified in Question 1?

- Very Hot
- Hot
- Warm
- Slightly Warm
- Neutral
- Slightly Cool
- Cool
- Cold
- Very Cold

**7:** How would you rate your overall Thirst on the day identified in Question 1?

- Not thirsty at all
- A little thirsty
- Moderately thirsty
- Very thirsty
- Very, very thirsty

**8:** How did you satisfy your thirst on the day identified in Question 1? Check all that apply.

- Thirst went away
- Drank water
- Drank juice/flavoured beverage
- Drank alcohol
- Other: __________

**9:** How did you manage the heat on the day identified in Question 1? Check all that apply.

- Stayed inside
- Reduced physical activity
- Used air conditioning
- Visited a cool place (i.e. mall, library, community centre, etc.)
- Stayed hydrated
- Cold bath/shower
- Other: ___________
